# Cascading signaling pathways improve the fidelity of a stochastically and deterministically simulated molecular RS latch

**DOI:** 10.1186/1752-0509-3-72

**Published:** 2009-07-17

**Authors:** Evan Mills, Kevin Truong

**Affiliations:** 1 Institute of Biomaterials and Biomedical Engineering, University of Toronto, 164 College Street, Toronto, Ontario, M5S 3G9, Canada; 2 Edward S. Rogers Sr. Department of Electrical and Computer Engineering, University of Toronto, 10 King's College Circle, Toronto, Ontario, M5S 3G4, Canada

## Abstract

**Background:**

While biological systems have often been compared with digital systems, they differ by the strong effect of crosstalk between signals due to diffusivity in the medium, reaction kinetics and geometry. Memory elements have allowed the creation of autonomous digital systems and although biological systems have similar properties of autonomy, equivalent memory mechanisms remain elusive. Any such equivalent memory system, however, must silence the effect of crosstalk to maintain memory fidelity.

**Results:**

Here, we present a system of enzymatic reactions that behaves like an RS latch (a simple memory element in digital systems). Using both a stochastic molecular simulator and ordinary differential equation simulator, we showed that crosstalk between two latches operating in the same spatial localization disrupts the memory fidelity of both latches. Crosstalk was reduced or silenced when simple reaction loops were replaced with multiple step or cascading reactions, showing that cascading signaling pathways are less susceptible to crosstalk.

**Conclusion:**

Thus, the common biological theme of cascading signaling pathways is advantageous for maintaining the fidelity of a memory latch in the presence of crosstalk. The experimental implementation of such a latch system will lead to novel approaches to cell control using synthetic proteins and will contribute to our understanding of why cells behave differently even when given the same stimulus.

## Background

Biological systems have been compared with digital systems, but such comparisons cannot be stretched too far as biological systems are affected by crosstalk between signals due to diffusivity in the medium, reaction kinetics and geometry [1-7]. Biological systems share some similarities with digital systems, such as basic logic functions and emergent network properties (i.e. memory and robustness) [1-4,6,7]. As demonstration, logic functions (AND, OR and NOT gates) have been constructed out of many biological molecules, such as DNA and proteins [1,5,6]. However, the analogy is limited by the basic issue of connectivity. Digital systems communicate between constituent parts using wires, providing direct connections between components with minimal interference. With biological systems, however, the biomolecule must diffuse through a common medium to find its binding partner, without confusing it with another similar partner. When biomolecular binding interactions are "confused" in this way, the result is crosstalk between distinct systems and pathways.

**Figure 1:**
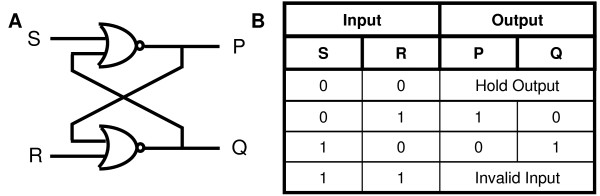
**Schematic diagram of an RS Latch**. **A**, The RS latch is created using two cross coupled NOR gates. **B**, Truth table for a NOR gated RS latch. Note that the case where both inputs are logic 1 is not allowed.

Demonstrating a memory element using biological components will be an important milestone to understanding how complex behaviours evolve in biological systems. The analogous development of memory elements in digital systems have allowed the creation of finite state machines (FSMs) that can respond to external inputs based on the memory of the system's state TAS notedsdfjkldfjkldhe most basic memory element in digital systems is the RS latch which can be created using two cross coupled NOR gates (Figure 1A). Parallel to this, memory can exist in cells in two contexts: a global, FSM-like state or a local, RS latch-like state. A well studied system of global memory in a cellular context is the cell cycle [8,9]. An example of memory storage in this context is the differential response of a cell to the protein synthesis drug cycloheximide. A pre-restriction point cell will not divide if treated with cycloheximide while a post-restriction point cell will carry on with cell division in the presence of the drug [9]. This shows that the restriction point is an example of a global cellular memory state, where a cell's memory has altered its response to an environmental stimulus. Local units of cellular memory are often considered to be small enzyme networks such as kinase or phosphorylase feedback loops [10]. While the local memory mechanisms in a cell may not resemble the cross coupled NOR gate configuration of an RS latch, it may have an equivalent memory behaviour that is described by the truth table of the RS latch behaviour (Figure 1B).

Crosstalk between proteins and signaling pathways introduces complexity and flexibility to cellular systems. However in the context of memory storage, there must be a high degree of fidelity between input signals and the various components of a system [8]. Many enzymes have broad ranges of binding affinity for different substrates that allows them to affect a variety of cellular pathways. For example, receptor tyrosine kinases (RTKs) represent a family of enzymes that respond to different upstream signals (epidermal, fibroblast and platelet-derived growth factors), and yet can all result in the activation of the GTPase Ras to a varying degree [11]. Consequently, at least three different input signals can all interact with the same effector protein producing crosstalk between various downstream pathways. However, it is undesirable for a memory system to be affected by molecules which are not being directly controlled by the desired, specific, upstream signal [8].

A stochastic biomolecular simulator based on previous models [12-22], as well as an ordinary differential equation simulator [13], was used to investigate a simple network of proteins that can replicate the functionality of an RS latch in the context of crosstalk. We chose to simulate crosstalk reactions using both methodologies because while classical deterministic modeling based on differential equations can efficiently simulate simple systems, their assumptions of spatial and temporal homogeneity are not always accurate in dynamic biological systems. Furthermore, in some systems the number of molecules being considered can be very low, thus resulting in a substantial degree of noise in a reaction network which cannot easily be handled with differential equations. In contrast, stochastic modeling can address both issues by allowing for the random movement of individual molecules in a particular location [1,23]. Stochastic processes in biological systems modeling initially focused on gene expression [24-28], but have also been studied in other pathways such as metabolism and mitosis [29-32]. Thus, crosstalk was modeled as a set of enzymatic reactions between two RS latch systems. An enzyme from one RS latch system could react with the substrate of an analogous enzyme from the second system, but the reaction occurred at a reduced rate, usually 0.1 to 0.01 that of the analogous enzyme [4].

## Results and discussion

### RS latch behaviour resembles enzymatic reactions

**Figure 2:**
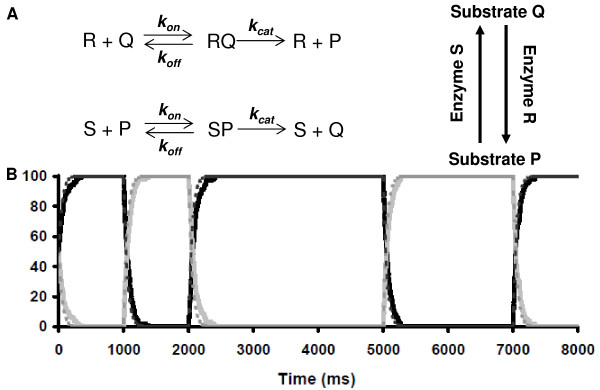
**Implementation and simulation of a molecular RS latch**. **A**, Biochemical reactions used to generate latch behaviour (left) with the representation as an inverse enzymatic cycle (right). **B**, Simulated time course of the RS latch system using stochastic (solid lines, number of molecules) and deterministic (dashed lines, concentration in millimolar) simulations with specie Q in dark grey lines and P in light grey lines. At t = 0 ms, an equal amount of both substrates is present. The amount of an enzyme was either 20 molecules (stochastic) or 20 mM (deterministic); enzyme S is added at t = 0 ms, replaced with R at 1000 ms, S is returned at 2000 ms, all enzymes are removed at 3000 ms, R is returned at 5000 ms and removed at 6000 ms, and finally S is returned at 7000 ms.

A simple reaction system was created with the behaviour of an RS latch described by its truth table (Figure 2). In this form the RS latch is essentially a bistable chemical reaction network [10]. Enzyme R converts substrate Q to P, while enzyme S converts substrate P to Q. Thus, in the sole presence of enzyme R, substrate P will eventually dominate, while in the sole presence of enzyme S, substrate Q will eventually dominate. In the absence of both enzymes S and R, substrate concentrations do not change and the last dominate substrate is remembered. Through a combination of association and dissociation reactions, the reaction system described above was created (Figure 2A). The reaction rates, *k*_*on*_, *k*_*off*_, and *k*_*cat*_, as well as spatial constants such as volume, diffusivity and molecule size in the stochastic simulations are summarized in Table 1. Rate constants (*k*_*on*_, *k*_*off*_, and *k*_*cat*_) were based on a range of kinases and phosphatases from the mitogen activated protein kinase (MAPK) pathway [33], and as such our model of an RS latch is comparable to real biological systems. The result of this simulation is identical to the ideal expected outcome, except for the presence of a time constant (Figure 2B).

**Table 1:** Constants and values used in stochastic simulations

Constant	Meaning	Value	Units
*k*_*on*_	Association rate	10^4^	M^-1^s^-1^
*k*_*off*_	Dissociation rate	10^0^	s^-1^
*k*_*cat*_	Catalysis rate	10^4^	s^-1^
V	Volume of reaction space		m^3^
m_enzyme_	Mass of enzyme	10^4^	gmol^-1^
m_substrate_	Mass of substrate	10^3^	gmol^-1^
D	Diffusion coefficient	10^-10^	m^2^s^-1^

Rate constants were based on kinases and phosphatases from the MAPK pathway, retrieved from the Database of Quantitative Cellular Signalling [33].

An RS latch implemented in this way can be a model of a pair of inverse enzymatic reactions. That is, the enzyme R can be a kinase that phosphorylates the substrate Q to phospho-Q, or substrate P. The enzyme S is then a phosphorylase that converts substrate P back to substrate Q. In this way, the simplest biological memory unit is the functional state (in this model, the phosphorylation state specifically) of a particular molecule. More complex states, such as phenotypes, can be built up of many molecular states in the same way an FSM is built of many RS latches. In the biological context, it could be possible for both a phosphorylase and kinase to be available to act on a substrate at the same time. However, this conflicts with the invalid input R = S = 1 for an RS latch. Going forward, we will assume that upstream signals that control phosphorylation and dephosphorylation will suppress this possibility thereby maintaining a clearly defined input state for our model.

### Crosstalk requires modification to latch design

**Figure 3:**
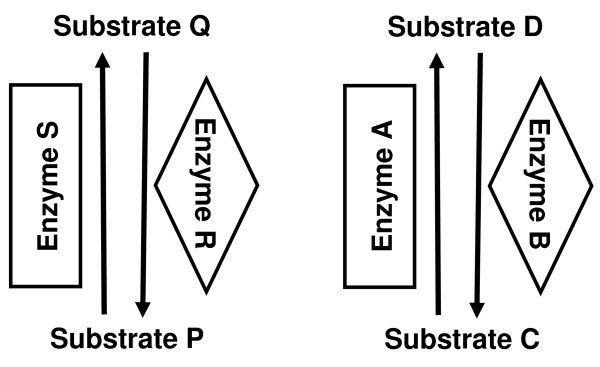
**Schematic of crosstalk interactions between two RS latches in the same space**. Enzymes enclosed by the same shapes (rectangles or diamonds) can have crosstalk interactions. Specifically, Enzyme S like enzyme A can catalyze substrate C to D, although at a slower rate than enzyme A. Enzyme A can similarly catalyze substrate P to Q at a slower rate than enzyme S. The same relationship is true for enzymes R and B.

**Figure 4:**
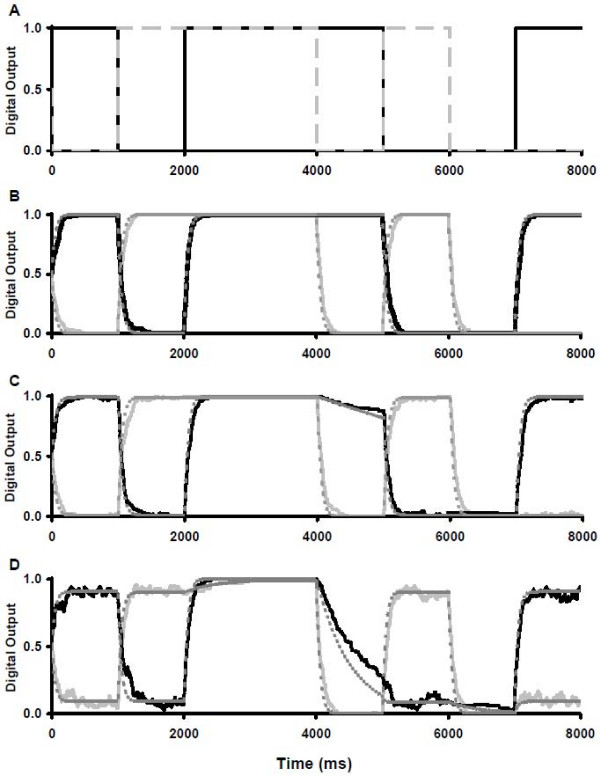
**Simulation of two RS latches with varying degrees of crosstalk**. **A**: Expected output of two ideal RS latches (substrate P is light grey, C is dark grey), **B**: biochemical latches with no crosstalk, **C**: minimal crosstalk, and **D**: moderate crosstalk. Stochastic results (solid lines) are superimposed with deterministic results (dashed lines) for panels B-D with substrates P (light lines) and C (dark lines). The relative values have been normalized to range between 0 and 1 as in a digital RS latch. For all panels the inputs were as follows: At t = 0 ms, S = 1, B = 1; at 1000 ms, R = 1, A = 1; at 2000 ms, R = 1, B = 1; at 3000 ms, A = B = S = R = 0; at 4000 ms, S = 1, A = B = 0; at 5000 ms, R = 1, A = 1; at 6000 ms, S = 1, A = 1; at 7000 ms, S = 1, B = 1.

As the crosstalk between two latches in the same spatial location increased, the fidelity of each latch decreased (Figures 3 and 4). In this example, a system of two latches was simulated, where the enzymes enclosed in the same shapes (rectangle or diamond) can crosstalk with each other's substrates (Figure 3). That is, enzyme A associates with substrate P at a reduced rate of either 0.1 or 0.01 that of enzyme S; that is, *k*_*on *_is reduced for the crosstalk reactions. Enzyme S similarly associates with the substrates of enzyme A. The two enzymes in diamond shaped boxes (enzymes R and B) behave in the same way with respect to each other. A simulation with different inputs and expected outputs for each latch was performed and one output population of molecules per latch was tracked each time (substrate P for SRPQ and substrate C for ABCD).

To evaluate the effect of crosstalk between latches and our attempts to silence it, simulations were performed throughout the paper under three scenarios, each compared with the ideal output (Figure 4A): no crosstalk (Figure 4B), minimal crosstalk with k_crosstalk _= 0.01k_primary _(Figure 4C) and moderate crosstalk with k_crosstalk _= 0.1k_primary _(Figure 4D). Under the no crosstalk scenario, each latch behaved ideally (Figure 4B). The continued presence of a non-zero time constant is a physical reality and cannot be eliminated. The initial conditions were set to a random state, represented by each output being 50% of its maximal potential output. When the first input was provided (S = 1 and B = 1), the outputs quickly approached their expected values of P = 0 and C = 1. Once a value was achieved, it was maintained until the next input signal arrived at 1000 ms.

Under the minimal crosstalk scenario, there were three important changes in the system's behaviour, which were revealed by one or both simulation methods (Figure 4C). First, the output signals P and C did not reach their new values as quickly (both methods) or as smoothly (stochastic method only) as with no crosstalk. Second, once the steady state value for P or C was achieved, it was not as complete a signal as with no crosstalk (both methods) and there was fluctuation in the output (stochastic method only). With both simulation methods, the desired output for the latches from time points 0 to 1000 ms were no longer P = 0 and C = 1 but rather approximately P = 0.02 and C = 0.98. The fluctuations in output were evident in only the stochastic simulations: for example, from time points 0 ms to 2000 ms. During this time period, the latches were assuming opposite values. As a result, once one latch achieved close to 100% of its desired output, an opposing enzyme from the other latch could temporarily alter a substrate into the undesired state. The temporal nature of this interaction was lost in the deterministic modeling. A third consequence of crosstalk was a partial loss of the substrate C signal between 4000 ms and 5000 ms because one system is passively holding its output while the other is actively outputting the opposite value (both methods). In this period one system, SRPQ, was being actively set to the substrate P = 0 state while the ABCD system was in the "hold output" state for substrate C = 1. Essentially, the SRPQ system was overriding the information that was stored in the ABCD system.

Under the moderate crosstalk scenario, the latches showed an exaggeration of the trends established in the minimal crosstalk example, such as reduced steady state signal (now P = 0.10 and C = 0.90) and output fluctuations (Figure 4D). Notably, the loss of substrate C signal from 4000 ms to 5000 ms increased. In this situation, substrate C dropped below 0.5 and so the ABCD system outputted substrate D = 1 by the end of the relevant time period. Hereafter, this phenomenon will be referred to as "forced state switching". This is a serious flaw in the latch system and must be corrected to ensure a high fidelity memory system. To improve the memory storage fidelity of the two latch systems, any solution must reduce forced state switching and the fluctuations in output values.

### Reaction modifications to silence the effect of crosstalk

**Figure 5:**
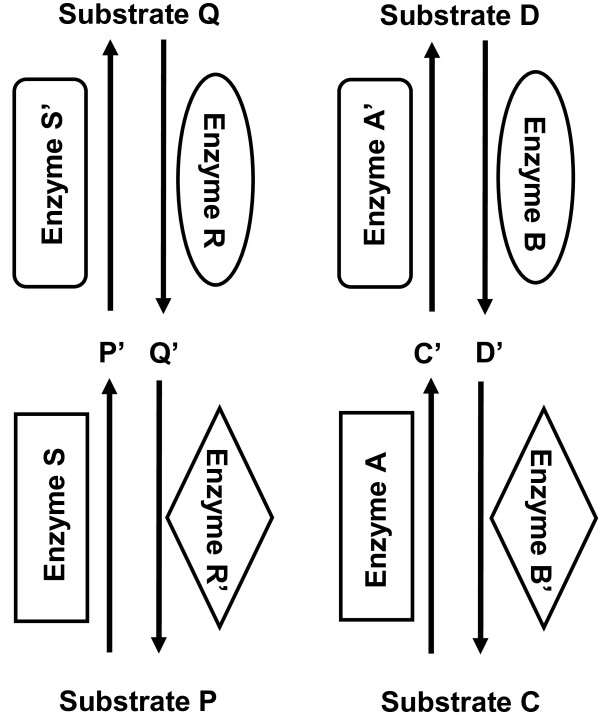
**Schematic diagram of crosstalk between a pair of two-step latches**. Each latch has been extended to include two enzymes per reaction cascade with one intermediate. Crosstalk is implemented between enzymes enclosed in similar shapes, as before. This can be further extended to a three step loop.

**Figure 6:**
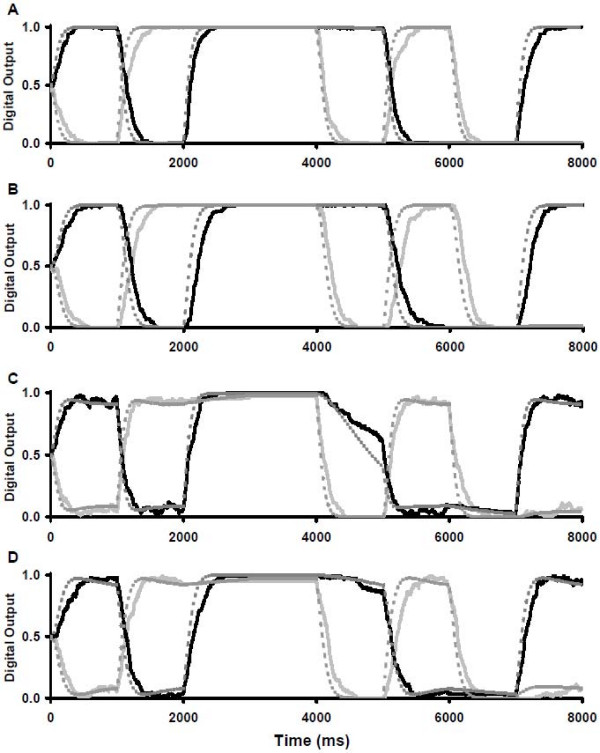
**Simulation of two RS latches with varying degrees of crosstalk and cascading signaling pathways**. **A**: Two steps with minimal crosstalk, **B**: Three steps with minimal crosstalk **C**: Two steps with moderate crosstalk, and **D**: Three steps with moderate crosstalk. The same inputs were provided as for the simulations of Figure 4. Stochastic results (solid lines) are superimposed with deterministic results (dashed lines) for substrates P_T _(light lines) and C_T _(dark lines). Results have been scaled to range between 0 and 1 as in a digital RS latch. Outputs have been redefined such that P_T _= P + P' for two step loops or P_T _= P + P' + P" for three steps, and similarly for other outputs.

Introducing a multiple step or cascading pathway to each latch improved overall fidelity and the addition of more steps further improved the fidelity but required more time to reach a steady state (Figures 5 and 6). Inspired by biological signaling pathways, the simple reaction system of the RS latch was extended by one and two steps in an enzyme cascade fashion (Figure 5). An extended cascade for controlling protein activation frequently occurs in signaling pathways. For example, the mitogen activated protein kinase (MAPK) pathway is a conserved signaling pathway that culminates with MAPK activation. MAPK activation can only occur once threonine and tyrosine residues in the TXY loop of MAPK are phosphorylated [34,35]. This is accomplished by upstream MEK kinases in the MAPK pathway. This represents a situation where a protein must undergo two separate transformations to become activated. Similarly, to completely return MAPK to its original, inactive state, both the threonine (Thr) and tyrosine (Tyr) residues must be dephosphrylated by serine/threonine phosphatase PP2A and tyrosine phosphatase PTP, respectively [35]. This situation is analogous to our two step latch where inactive MAPK is P, active MAPK is Q, upstream kinases are S and S' and phosphatases are R and R', while P' and Q' represent intermediate states of MAPK.

The crosstalk in a pair of two-step latches was implemented similarly to the original one step latch (Figure 5). Now, there was additional crosstalk between enzymes S' and A', shown in rounded rectangles, and the enzymes R' and B', shown in ellipses. Crosstalk is biologically motivated using the same principles as with a one-step loop: phosphorylation and dephosphorylation can be catalyzed by a range of kinases and phosphatases on a variety of substrates with different kinetics. This idea is easily extrapolated for a three-step latch.

Under the minimal crosstalk scenario, a pair of two-step (Figure 6A) or three-step latches (Figure 6B) eliminated the effect of crosstalk by reducing fluctuations and minimizing forced state switching (previously observed in Figure 4B). However, the time constant increased considerably when compared with the no crosstalk scenario using a one-step latch (Figure 4A). These trends were noticeable in both the stochastic and deterministic simulations. Essentially, the second and third steps slowed the reaction by requiring more collisions between substrates and enzymes.

Under the moderate crosstalk scenario, the two-step latch showed some improvement in fidelity (Figure 6C), however the improvements were more noticeable with three steps (Figure 6D). In this scenario, significant improvement in fluctuations, steady state output and forced state switching was not seen until a third step was added, suggesting that cascading reaction pathways are able to transmit signals with a higher fidelity. Furthermore, the forced state switching from substrate C = 1 to substrate D = 1 between 4000 ms and 5000 ms was prevented (Figure 6D). However, there were two discrepancies between the simulation methods. First, the deterministic simulations showed an overshoot past the steady state value for both the two step and three steps loops that is not present with the stochastic simulations. Second, the deterministic simulations suggest that the three step loop is not as successful at restoring the steady state output as was suggested by the stochastic simulations (deterministic predicts C = 0.92 versus C = 0.96 for stochastic from time 0 to 1000 ms). These discrepancies could be due to the fluctuations present in the stochastic simulations that are not present in the deterministic simulations.

The effective output state of the system was re-defined for the multiple step latches: for the two-step loops, the outputs were the sum of substrates P + P', and similarly for the other substrates Q, C, and D; for three-step loops, the sum of substrates P + P' + P". Essentially, the additional steps in the latch buffered the output molecules and limited the effect of the crosstalk not on any one molecule, but the reaction pathway as a whole. Thus, each of the molecular species was summed because it was part of the same pathway and had a similar functionality. To see a biological basis for this, consider the MAPK example again. Three distinct molecules make one half of the reaction pathway (the Q, Q' and Q" half): unphosphorylated MAPK, p-Tyr MAPK and p-Thr MAPK. All three molecules represent inactive forms of MAPK, and so summing them represents an overall state rather than a specific molecule. The summing operation is a way of reconstructing the state from a collection of distinct molecules. The second, activated, state of MAPK is only achieved when MAPK is doubly phosphorylated [34,35], which is not included in the sum, and resides in the other half of the reaction loop. Given the model of a memory state as a molecule switching between its phosphorylated and de-phosphorylated states, it is likely that a molecule can only exist in two functional states (on or off). However, such a model could only apply to situations where the functionalities of each of the intermediate molecules in one half of the reaction loop are the same and would preclude a molecule being in a transition state where it was either partially active or had some functionality unrelated to either end state.

Both simulation methods showed that the time constant of the switch was longer for reactions with more steps (Figure 6). This suggested that cascading pathways had two competing factors: longer cascading pathways transmitted higher fidelity signals, but required more time to reach a steady state. This effect was more exaggerated in the stochastic simulations compared to the deterministic simulations even though the steady state values were similar. Given that the stochastic simulations provided more information on temporal details in previous simulations (Figure 4), it is likely that the stochastic simulation is showing a more accurate representation of the system in its early stages. The compromise between speed and other biological networks parameters has been noted in previous work such as negative feedback loops that reduce rise times at the expense of steady state signal [36] and reaction conditions that improve signal fidelity and specificity at the expense of speed using compartmentalization [8] or low-affinity scaffolding [37]. While speed is sacrificed in our simulations to improve fidelity, biological mechanisms exist that can speed the reaction network without loss of fidelity such as high-affinity scaffolding in the case of the MAPK cascade [38].

Overall, this work provides a basis for interpreting biochemical signaling cascades in terms of biological memory and the limitations placed on this by crosstalk. First, we have shown that it is possible to consider a very simple enzymatic system (any two complementary enzymes such as a kinase and phosphatase) as a memory storage unit based on the functional state of their common substrate. However, without modification, the initial memory storage system failed under conditions of crosstalk. Crosstalk, which is used by cells to both reduce the number of different proteins necessary for signaling cascades as well as to add complexity to signal regulation, has a significant parasitic effect on the storage of memory with even two systems present. We have shown that the parasitic effects of crosstalk can be silenced by cascading several components to create one long multistep cascade. This finding provides insight into the length of biological systems as well as the tradeoff between network parameters that we have discussed above. The ability of cells to store memory allows them to interpret future signals appropriately by combining these inputs with past information [10]. A model of biological memory will improve our ability to interpret how cells respond to signals as they do, and may provide insights into their rational manipulation.

## Conclusion

This study used stochastic and deterministic modeling to show that a system of two reciprocal enzymes that toggles a substrate between two states can resemble a memory element, specifically an RS latch. However in a cellular environment, crosstalk between similar enzymes receiving different upstream signals will likely interfere with any signal transduction or memory storage system. This was shown to be the case when only two latch systems were operated in the same spatial localization with a moderate degree of crosstalk. Increasing the number of steps in the latches improved the fidelity of two latch systems. However, additional steps also increased the switching time constant thus slowing the system response. This may provide a basis for explaining the length of biological reaction cascades as a compromise between demands for fidelity and speed of response. Further modeling of biological systems' outputs based on known inputs as well as current states stored in molecular memory will aid in understanding why cells behave differently even when given the same stimulus. Implementing such a latch system *in vitro *to prove its feasibility may also lead to novel approaches to cell control using synthetic proteins that can be made to behave as a memory system.

## Methods

### Stochastic and deterministic simulators used

A stochastic simulator was developed for this study based on previous stochastic simulators [12-22]. The stochastic simulator tracks the location of each molecule created and allows it to perform a random walk (diffuse) through space. Collisions between molecules are the basis of mass action reactions. The stochastic simulator accepts user scripts defining a set of molecules with a particular size and diffusion coefficient. Then, a set of reactions is modelled as some combination of association between two molecules with a rate, k_f_, or dissociation of one molecule into others with a rate, k_r_. The user can then specify time points for the addition or removal of molecules.

Deterministic simulations were carried out using Dynetica [13].

### Crosstalk modeling

Crosstalk was incorporated into the simulation as follows, based on previous definitions [4,7,8]. Crosstalk, essentially a type of interference, is only relevant when two or more systems are present. Assume that there are two systems, one termed SRPQ with four elements, S, R, P and Q and an identical one termed ABCD with elements A, B, C and D. Assume that in SRPQ there is one reaction, which is S + P → SP → S + Q at a rate k_primary _= 10^4^. In the second system there is an equivalent reaction A + C → AC → A + D at the same rate. Crosstalk is then incorporated as an additional layer between the two systems. The exact way that the crosstalk reactions are modeled depends on the nature of the model. For example, if S and A are enzymes, Q and D are substrates and P and C are reaction products, then the cross-talk reactions would be S + C → SC → S + D at a rate k_crosstalk _= 10^3 ^and A + P → AP → A + Q at the same reduced rate [4,7]. Usually k_crosstalk _= 0.1 k_primary _(moderate crosstalk) or 0.01 k_primary _(minimal crosstalk).

## Authors' contributions

EM carried out the simulations, data analysis and drafted the manuscript. KT conceived the study, provided direction and helped draft the manuscript. All authors have read and approved the final manuscript.

## References

[B1] Pham E Li I Truong K Computational modeling approaches for studying of synthetic biological networks Current Bioinformatics 2008 3 130 141 10.2174/157489308784340667

[B2] McAdams HH Shapiro L Circuit simulation of genetic networks Science 1995 269 650 656 10.1126/science.7624793 7624793

[B3] Milo R Shen-Orr S Itzkovitz S Kashtan N Chklovskii D Alon U Network motifs: simple building blocks of complex networks Science 2002 298 824 827 10.1126/science.298.5594.824 12399590

[B4] Bhalla US Iyengar R Emergent properties of networks of biological signaling pathways Science 1999 283 381 387 10.1126/science.283.5400.381 9888852

[B5] Unger R Moult J Towards computing with proteins Proteins 2006 63 53 64 10.1002/prot.20886 16435369

[B6] Bray D Protein molecules as computational elements in living cells Nature 1995 376 307 312 10.1038/376307a0 7630396

[B7] Weng G Bhalla US Iyengar R Complexity in biological signaling systems Science 1999 284 92 96 10.1126/science.284.5411.92 10102825 PMC3773983

[B8] Komarova NL Zou X Nie Q Bardwell L A theoretical framework for specificity in cell signaling Mol Syst Biol 2005 1 2005.0023 10.1038/msb4100031 16729058 PMC1681467

[B9] Novak B Tyson JJ A model for restriction point control of the mammalian cell cycle J Theor Biol 2004 230 563 579 10.1016/j.jtbi.2004.04.039 15363676

[B10] Ramakrishnan N Bhalla US Memory switches in chemical reaction space PLoS Comput Biol 2008 4 e1000122 10.1371/journal.pcbi.1000122 18636099 PMC2440819

[B11] Simon MA Receptor tyrosine kinases: specific outcomes from general signals Cell 2000 103 13 15 10.1016/S0092-8674(00)00100-8 11051543

[B12] Sedwards S Mazza T Cyto-Sim: a formal language model and stochastic simulator of membrane-enclosed biochemical processes Bioinformatics 2007 23 2800 2802 10.1093/bioinformatics/btm416 17855418

[B13] You L Hoonlor A Yin J Modeling biological systems using Dynetica – a simulator of dynamic networks Bioinformatics 2003 19 435 436 10.1093/bioinformatics/btg009 12584138

[B14] Le Novere N Shimizu TS STOCHSIM: modelling of stochastic biomolecular processes Bioinformatics 2001 17 575 576 10.1093/bioinformatics/17.6.575 11395441

[B15] Lok L Brent R Automatic generation of cellular reaction networks with Moleculizer 1.0 Nat Biotechnol 2005 23 131 136 10.1038/nbt1054 15637632

[B16] Hoops S Sahle S Gauges R Lee C Pahle J Simus N Singhal M Xu L Mendes P Kummer U COPASI – a COmplex PAthway SImulator Bioinformatics 2006 22 3067 3074 10.1093/bioinformatics/btl485 17032683

[B17] Boulianne L Al Assaad S Dumontier M Gross WJ GridCell: a stochastic particle-based biological system simulator BMC Syst Biol 2008 2 66 10.1186/1752-0509-2-66 18651956 PMC2517591

[B18] Mao L Resat H Probabilistic representation of gene regulatory networks Bioinformatics 2004 20 2258 2269 10.1093/bioinformatics/bth236 15073019

[B19] Meng TC Somani S Dhar P Modeling and simulation of biological systems with stochasticity In Silico Biol 2004 4 293 309 15724281

[B20] Sanford C Yip ML White C Parkinson J Cell++ – simulating biochemical pathways Bioinformatics 2006 22 2918 2925 10.1093/bioinformatics/btl497 17038347

[B21] Adalsteinsson D McMillen D Elston TC Biochemical Network Stochastic Simulator (BioNetS): software for stochastic modeling of biochemical networks BMC Bioinformatics 2004 5 24 10.1186/1471-2105-5-24 15113411 PMC408466

[B22] Ohki N Hagiwara M Bio-Object, a stochastic simulator for post-transcriptional regulation Bioinformatics 2005 21 2478 2487 10.1093/bioinformatics/bti316 15705653

[B23] Gillespie DT Stochastic simulation of chemical kinetics Annu Rev Phys Chem 2007 58 35 55 10.1146/annurev.physchem.58.032806.104637 17037977

[B24] Elowitz MB Leibler S A synthetic oscillatory network of transcriptional regulators Nature 2000 403 335 338 10.1038/35002125 10659856

[B25] Blake WJ M KA Cantor CR Collins JJ Noise in eukaryotic gene expression Nature 2003 422 633 637 10.1038/nature01546 12687005

[B26] Elowitz MB Levine AJ Siggia ED Swain PS Stochastic gene expression in a single cell Science 2002 297 1183 1186 10.1126/science.1070919 12183631

[B27] McAdams HH Arkin A Stochastic mechanisms in gene expression Proc Natl Acad Sci USA 1997 94 814 819 10.1073/pnas.94.3.814 9023339 PMC19596

[B28] Weinberger LS Burnett JC Toettcher JE Arkin AP Schaffer DV Stochastic gene expression in a lentiviral positive-feedback loop: HIV-1 Tat fluctuations drive phenotypic diversity Cell 2005 122 169 182 10.1016/j.cell.2005.06.006 16051143

[B29] Arkin A Ross J McAdams HH Stochastic kinetic analysis of developmental pathway bifurcation in phage lambda-infected Escherichia coli cells Genetics 1998 149 1633 1648 9691025 10.1093/genetics/149.4.1633 PMC1460268

[B30] Borisuk MT Tyson JJ Bifurcation analysis of a model of mitotic control in frog eggs J Theor Biol 1998 195 69 85 10.1006/jtbi.1998.0781 9802951

[B31] Chen KC Csikasz-Nagy A Gyorffy B Val J Novak B Tyson JJ Kinetic analysis of a molecular model of the budding yeast cell cycle Mol Biol Cell 2000 11 369 391 10637314 10.1091/mbc.11.1.369 PMC14780

[B32] Edwards JS Ibarra RU Palsson BO In silico predictions of Escherichia coli metabolic capabilities are consistent with experimental data Nat Biotechnol 2001 19 125 130 10.1038/84379 11175725

[B33] Sivakumaran S Hariharaputran S Mishra J Bhalla US The Database of Quantitative Cellular Signaling: management and analysis of chemical kinetic models of signaling networks Bioinformatics 2003 19 408 415 10.1093/bioinformatics/btf860 12584128

[B34] Zhang YY Mei ZQ Wu JW Wang ZX Enzymatic activity and substrate specificity of mitogen-activated protein kinase p38 alpha in different phosphorylation states Journal of Biological Chemistry 2008 283 26591 26601 10.1074/jbc.M801703200 18669639 PMC3258911

[B35] Camps M Nichols A Arkinstall S Dual specificity phosphatases: a gene family for control of MAP kinase function FASEB J 2000 14 6 16 10627275

[B36] Rosenfeld N Elowitz MB Alon U Negative autoregulation speeds the response times of transcription networks J Mol Biol 2002 323 785 793 10.1016/S0022-2836(02)00994-4 12417193

[B37] Bardwell L Zou X Nie Q Komarova NL Mathematical models of specificity in cell signaling Biophys J 2007 92 3425 3441 10.1529/biophysj.106.090084 17325015 PMC1853162

[B38] Zou X Peng T Pan Z Modeling specificity in the yeast MAPK signaling networks J Theor Biol 2008 250 139 155 10.1016/j.jtbi.2007.09.024 17977559

